# Epipericardial Fat Necrosis in COVID‐19—A Case Report

**DOI:** 10.1002/ccr3.72179

**Published:** 2026-02-26

**Authors:** Klaric Ivana, Zovko Tanja, Goluza Gordana, Pavlovic Katica, Berberovic Marina, Pravdic Nikolina, Goluza Sesar Marija

**Affiliations:** ^1^ Center for Emergency Medicine University Clinical Hospital Mostar Mostar Bosnia and Herzegovina; ^2^ Faculty of Medicine University of Mostar Mostar Bosnia and Herzegovina; ^3^ Department of Lung Disease and Tuberculosis University Clinical Hospital Mostar Mostar Bosnia and Herzegovina; ^4^ Clinic for Urology University Clinical Hospital Mostar Mostar Bosnia and Herzegovina; ^5^ Clinic for Neurology University Clinical Hospital Mostar Mostar Bosnia and Herzegovina

**Keywords:** acute chest pain, case report, COVID‐19 infection, epipericardial fat necrosis

## Abstract

Epipericardial fat necrosis (EFN) is a rare but benign cause of acute chest pain that can mimic life‐threatening conditions such as myocardial infarction, pulmonary embolism, and aortic dissection. This case highlights the importance of recognizing EFN to prevent unnecessary interventions.

## 1 Introduction

Epipericardial fat necrosis (EFN) is an inflammatory process that affects the mediastinal fat surrounding the heart [1]. Epicardial adipose tissue is the adipose tissue located between the myocardium and the visceral pericardium and moves with the heart, while pericardial adipose tissue is static. It is situated around the pericardium, and they jointly form the pericardial sac [2]. EFN is a rare cause of chest pain that can be confused with acute, life‐threatening conditions such as pulmonary embolism, myocardial infarction, aortic dissection, acute pericarditis [1, 3] and other non‐cardiac causes of chest pain such as gastroesophageal reflux disease and costochondritis [4]. EFN has been reported in 0.26% of 7463 Multislice Computed Tomography (MSCT) of chest examinations and 2.15% of 926 patients who underwent chest MSCT because of atypical chest pain in the emergency department [5]. It is most frequently discovered incidentally on MSCT scan as part of the evaluation of acute chest pain [6, 7]. EFN is more common in men than in women, and the mean age of patients is 42.7 ± 13.6 years. It is significantly more common in the left hemithorax [5]. The EFN triad is based on CT and clinical findings and includes acute chest pain, an encapsulated fatty lesion with dense strands, and thickening of the pericardium [8].

The differential diagnosis of EFN based on imaging findings includes diaphragmatic hernia and fat‐containing tumors, such as lipoma, liposarcoma, and thymolipoma. The presence of stranding and rim surrounding the fatty lesion, intrinsic fat stranding of the lesion, peripheral rim enhancement, lack of solid components, and diaphragmatic integrity are effective in distinguishing EFN from other diseases. EFN is a benign, inflammatory process, not cancer, so tumor markers are not part of the routine diagnostic procedure [6, 9, 10]. Patients typically arrive at the emergency department with severe chest pain that worsens with deep inspiration [11]. The symptoms can lead to confusion in the working diagnosis, often leading to aggressive laboratory and radiological workup [12].

During the coronavirus disease (COVID‐19) pandemic, emergency departments were faced with an increased number of patients with chest pain, dyspnea, and nonspecific systemic symptoms, making rapid triage and accurate diagnosis even more difficult [13]. Although most reported cases of EFN were described independently of COVID‐19 or as an incidental radiological diagnosis, recent literature shows that EFN can also occur as part of active SARS‐CoV‐2 infection [14]. During the COVID‐19 pandemic, pericardial adipose tissue was susceptible to COVID‐19 due to the expression of the angiotensin‐converting enzyme 2 (ACE2) receptor [15]. This may be associated with EFN [14].

In this case report, we described a patient with severe left chest pain that was clinically presented during COVID infection and was a consequence of the EFN.

## 2 Case History/Examination

A 53‐year‐old female patient came to the Emergency Medicine Center for an examination due to severe pain in the left side of the chest, which lasted for three days. The pain was intense, appearing suddenly and intensifying with the slightest movement. The patient denied cough, expectoration, and difficulty breathing. She reported reduced exercise tolerance. The patient did not have any chronic or hereditary diseases. She had a nodular goiter of the thyroid gland without the need for therapy. The patient's family history was positive for malignant diseases (mother had colon cancer and father had lung cancer). She was a non‐smoker and did not use chronic therapy. Upon admission, vital signs were recorded and were within normal limits. Blood pressure was normal (RR 135/90 mmHg), heart rate was 85 beats per minute, and oxygen saturation was 98% on room air. The patient was afebrile and denied fever. A physical examination, including the cardiovascular, respiratory, neurological, and gastrointestinal systems, showed no significant features.

## 3 Differential Diagnosis, Investigations, and Treatment

**FIGURE 1:**
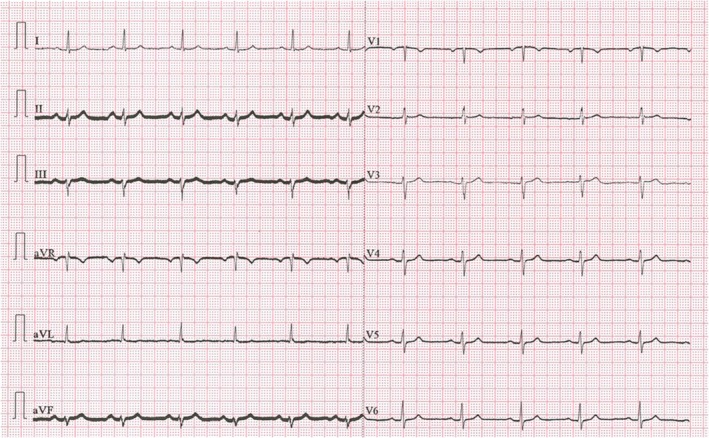
Electrocardiogram.

**FIGURE 2:**
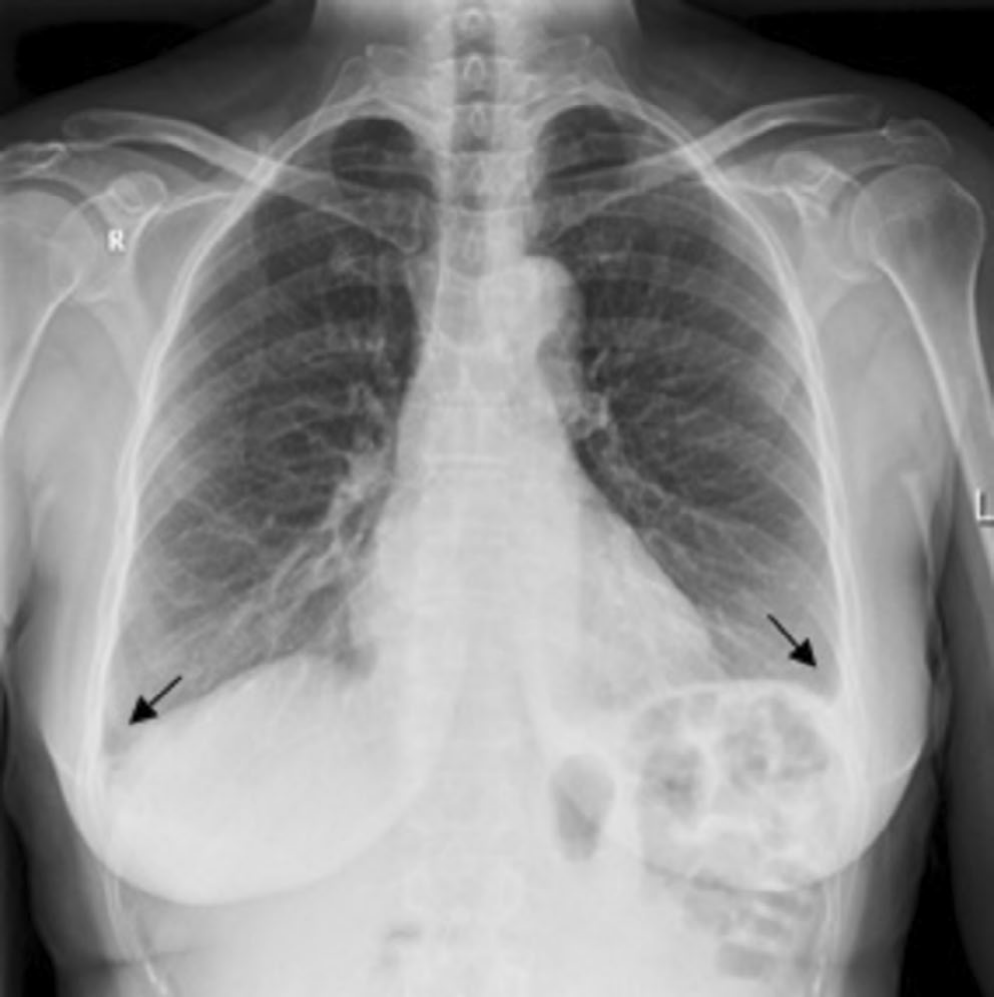
Frontal chest X‐ray on admission: Bilaterally visible smaller pleural effusions, more pronounced on the right.

**FIGURE 3:**
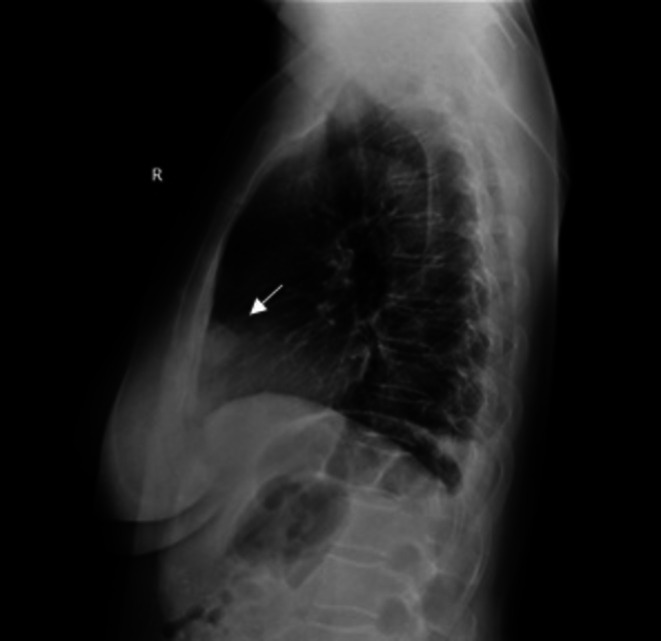
Lateral chest X‐ray on admission: Showed an oval, irregular shadow retrosternally.

**FIGURE 4:**
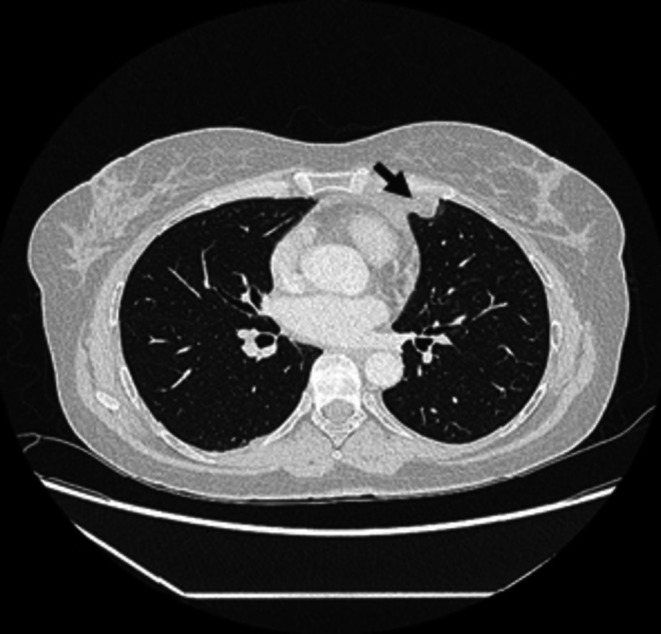
Multislice Computed Tomography of the chest Axial (Lung window) on admission: Visualized a mild lobulated, mostly hypodense infiltrate in the upper lung lobe of the left lung.

**FIGURE 5:**
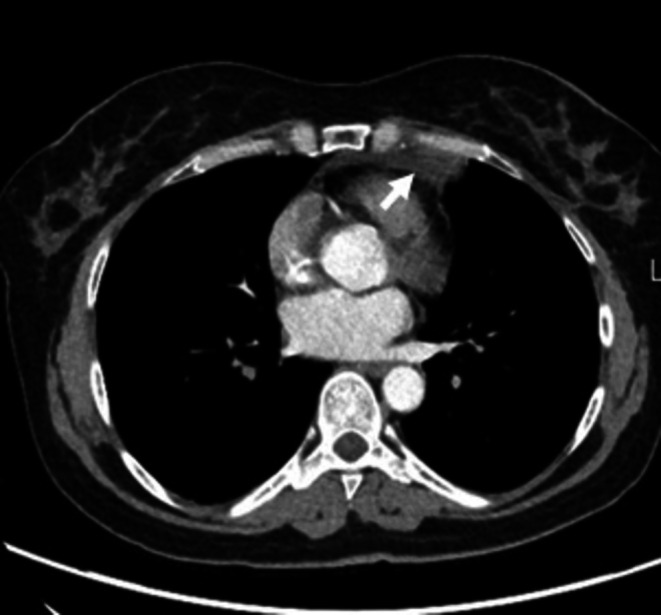
Multislice Computed Tomography of the chest Axial (Soft tissue window) on admission: Visualized a mild lobulated, mostly hypodense infiltrate in the upper lung lobe on the left lung.

**FIGURE 6:**
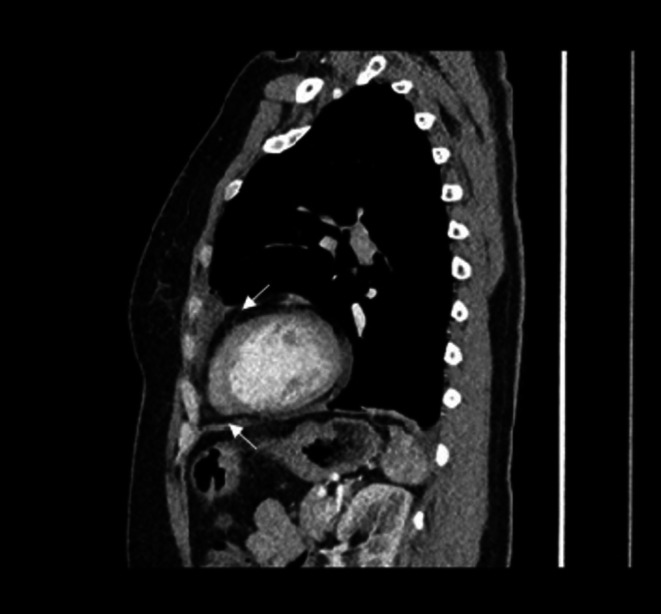
Multislice Computed Tomography of the chest Sagittal (Soft tissue window) on admission: Visualized a mild lobulated, mostly hypodense infiltrate in the upper lung lobe on the left lung.

A diagnostic work‐up was done. The electrocardiogram record was normal (Figure 1). The white blood count (WBC) was completely normal, C‐reactive protein (CRP) was slightly elevated at 15.1 mg/L (reference interval 0.0–5.0 mg/L), and high‐sensitive troponin was normal. The D‐dimer value was slightly elevated at 0.52 mg/L (reference interval < 0.5 mg/L) and was considered normal, considering the patient's age [8] (Table 1). Frontal and lateral chest X‐ray was performed (Figures 2 and 3), and showed an oval, irregular shadow retrosternally. After completing the initial diagnostic work‐up, the patient was hospitalized at the Department of Pulmonary Diseases and Tuberculosis for further diagnostic processing. Due to a complete diagnostic evaluation, the tumor markers were also performed, and all were within the reference interval. The MSCT of the chest visualized a mild lobulated, mostly hypodense infiltrate (LL with a diameter of about 20 mm; AP with a diameter of about 15 mm) in the upper lung lobe on the left lung, located next to the costal and mediastinal pleura; at the level of the costochondral junction of the 3rd and 4th ribs, which was suggestive of EFN. MSCT of the chest showed no signs of mediastinal lymphadenopathy, but showed a pericardial effusion ventrally next to the apex of the right ventricle with a width of about 10 mm. A minimal pleural effusion with a width of about 9 mm was visible at the left basal side (Figures 4, 5, 6). In addition, adhesive postural changes of the pleura and the presence of partial atelectasis along the left diaphragm were described. A transthoracic echocardiography (TTE) showed a pericardial effusion ventrally next to the apex of the right ventricle with a width of about 10 mm. As part of the further work‐up, the patient underwent abdominal ultrasound and neck ultrasound, which were normal. In the control laboratory, findings performed on the 5th day of hospitalization showed that the inflammation parameters were within the reference interval.

**TABLE 1:** Laboratory findings.

Investigation	Value	Reference range with unit
WBC	6.4	3.4–9.7 × 10^9^/L
Neutrophyli	4.00	2.06–6.49 × 109/L
Hemoglobin	134	138–175 g/L
Creatinine	51	64–104 μmol/L
Sodium	240	137–146 mmol/L
Potassium	3.8	3.9–5.1 mmol/L
Chloride	108	97–108 mmol/L
CRP	15.1	0.0–5.0 mg/L
AST	19	11–38 U/L
ALT	19	12–48 U/L
LDH	177	124–241 U/L
D‐dimer	0.52	< 0.50 mg/L
hs‐cTnI	0.3	< 15.6 pg/mL

Abbreviations: ALT, alanine aminotransferase; AST, aspartate aminotransferase; CRP, C‐reactive protein; GGT, gamma‐glutamyl transferase; hs‐cTnI, high sensitive troponin‐I; LDH, lactic acid dehydrogenase; WBC, white blood cell.

On the eighth day of hospitalization, the patient developed low‐grade fever. A PCR (polymerase chain reaction) test for COVID‐19 was performed. It came back positive, after which the patient was discharged for home treatment with a recommendation of home self‐isolation and a short course of the nonsteroidal anti‐inflammatory drug with ibuprofen 600 mg twice daily, and symptoms retreated within two weeks.

## 4 Conclusion and Results

**FIGURE 7:**
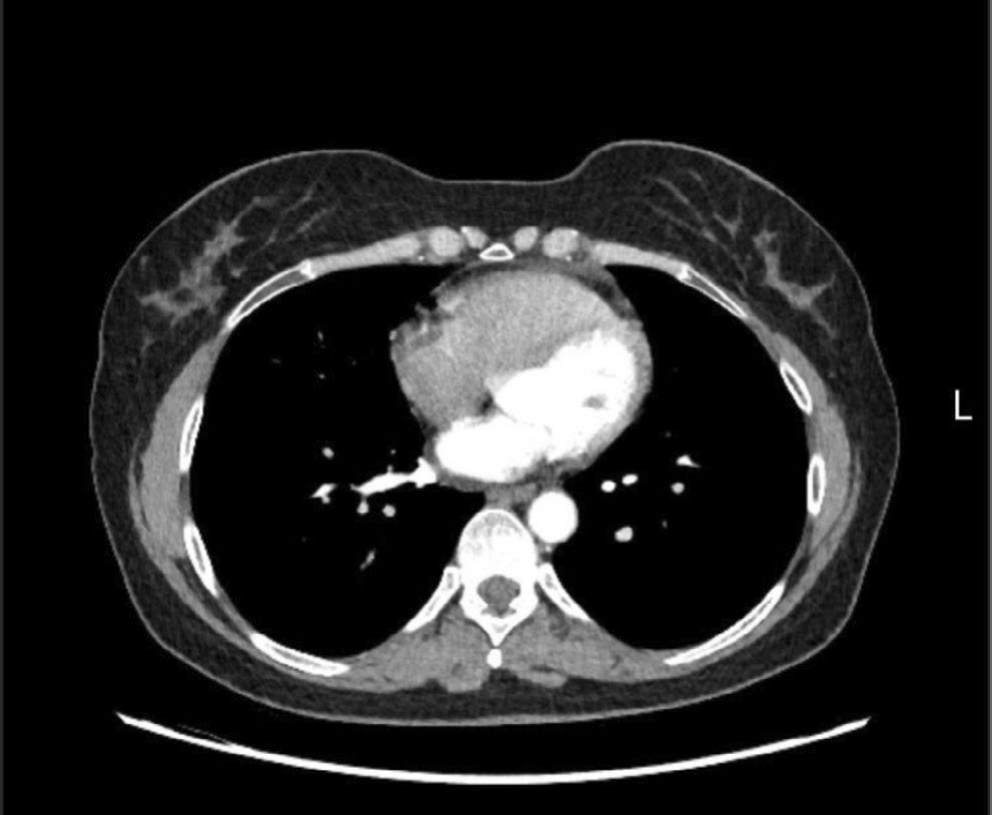
Control Multislice Computed Tomography of the chest Axial (Soft tissue window) after three months: Normal findings.

**FIGURE 8:**
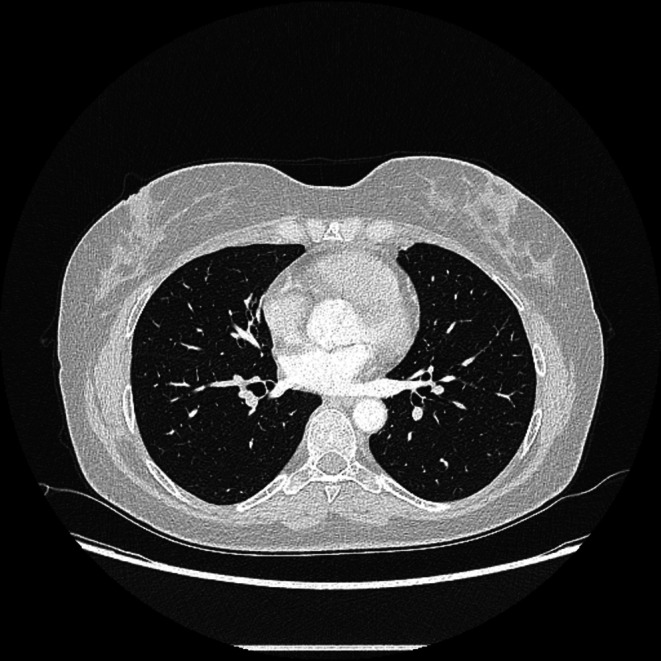
Control Multislice Computed Tomography of the chest Axial (Lung window) after three months: Normal findings.

On a follow‐up MSCT scan of the chest, performed three months after the initial scan, the previously described change in the upper left lung lobe, along the costal and mediastinal pleura at the level of the costochondral junction of the 3rd and 4th ribs, was not found (Figures 7 and 8). The patient was feeling well, without any symptoms.

EFN is a rare benign disease that causes acute chest pain, which is often unrecognized and may mimic more serious pathology, emphasizing the need for careful diagnostic evaluation. Patients with active or recent COVID‐19 and severe chest pain may have indications of EFN, which requires a careful diagnostic approach in emergency centers to recognize this rare condition and avoid unnecessary interventions properly. Incorporating EFN into the differential diagnosis helps optimize resource allocation and increases patient safety, preventing misdiagnosis of a benign condition as a medical emergency.

## 5 Discussion

EFN is a rare and self‐limiting cause of acute chest pain [16]. The first case of epipericardial fat necrosis in three patients was reported in 1957 [17]. Due to the pathological similarity of fat necrosis, it was thought that visceral fat necrosis was caused by torsion of the tissue itself, as occurs in intestinal appendages [18]. Approximately 70%–90% of EFN cases are misdiagnosed by clinicians as other diseases [19]. It can be detected in 2.2%–2.8% of chest MSCT scans performed for acute chest pain [5, 20]. Chest MSCT is an effective method for diagnosing EFN. Key MSCT signs include an oval or round lesion of fat density (−50 to −100 HU), sometimes with a wider range of values due to necrosis and inflammatory reaction, and perifocal inflammatory reaction, and sometimes thin edge density [5]. The insufficiently researched pathogenesis and intense clinical presentation of this benign condition have resulted in a wide range of diagnostic and treatment options over the decades. However, over time, many studies and case reports led to the conclusions we have now [16].

In our case report, we would like to highlight the symptoms as a triad: Severe pain on the left side of the chest, ipsilateral pleural effusion, and visible fat necrosis in the area of the heart fat tissue on the MSCT scan of the chest. It should be emphasized that, in comparison with laboratory tests (CRP, leukocytes, and D‐dimer), which are normal in this condition, as well as the patient's normal vital parameters, should raise suspicion of the EFN among other diagnoses. A short course of nonsteroidal anti‐inflammatory drugs provides symptom relief [16]. Follow‐up imaging is done to exclude underlying benign tumors (lipoma or hamartoma).

In the literature, there is currently very little evidence of recent or concomitant infection in patients with EFN [21]. We described a case of EFN in a patient with concomitant COVID‐19. During the COVID‐19 pandemic, emergency departments were faced with an increased number of patients with chest pain, dyspnea, and nonspecific systemic symptoms. That made rapid triage and accurate diagnosis even more difficult [13]. COVID‐19, being known as a respiratory illness, did not have a fully explained mechanism of action to elaborate on its effect on other organs [22].

To our knowledge, only a few cases of COVID‐19‐induced EFN have been reported in the literature previously. The mechanism underlying such inflammatory condition might be explained by the fact that epicardial adipose tissue, having the biggest proportions of lipogenesis and fatty acid metabolism among the visceral fat depots thus displaying thermogenic, mechanical and metabolic properties, is a target through ACE2 and the inflammatory cytokines interleukin‐6 (IL‐6), tumor necrosis factor (TNF) α which are expressed at high levels across this tissue. Subsequently, such interaction promotes an augmented cascade of inflammatory processes leading to myocardial and surrounding structures complications, including EFN [23]. EFN can be confidently diagnosed using imaging findings without the need for histological confirmation, providing important insights into its management in the emergency setting [24]. In our patient, tumor markers were obtained because of a positive family history of malignancy and the presence of an anterior mediastinal lesion on CT. Although not diagnostic on their own, normal values of multiple tumor markers helped us lower the suspicion of an underlying malignant process and supported the benign diagnosis of EFN [25].

While COVID‐19 was detected during hospitalization, the temporal relationship suggests that EFN may have preceded or coincided with subclinical infection. A causal link cannot be definitively established.

Although most reported cases of EFN have been described independently of COVID‐19 or as an incidental radiological diagnosis, recent literature shows that EFN can also occur in the context of active COVID‐19 infection [14]. Therefore, EFN in this context should not be considered exclusively a late sequela of COVID‐19, but also a possible complication during the acute illness.

## Author Contributions

**Klaric Ivana:** conceptualization, investigation, project administration, supervision, visualization, writing – original draft. **Zovko Tanja:** conceptualization, data curation, investigation, validation, writing – original draft. **Goluza Gordana:** methodology, supervision, validation, visualization. **Pavlovic Katica:** funding acquisition, methodology, resources. **Berberovic Marina:** funding acquisition, methodology, resources. **Pravdic Nikolina:** formal analysis, resources, software. **Goluza Sesar Marija:** data curation, formal analysis.

## Funding

The authors have nothing to report.

## Consent

Written informed consent was obtained from the patient for the publication of the case report.

## Conflicts of Interest

The authors declare no conflicts of interest.

## Data Availability

The data that support the findings of this study are available from the corresponding author upon reasonable request.
